# Genetic Background Affects Human Glial Fibrillary Acidic Protein Promoter Activity

**DOI:** 10.1371/journal.pone.0066873

**Published:** 2013-06-24

**Authors:** Xianshu Bai, Aiman S. Saab, Wenhui Huang, Isolde K. Hoberg, Frank Kirchhoff, Anja Scheller

**Affiliations:** Department of Molecular Physiology, University of Saarland, Homburg, Germany; University of Durham, United Kingdom

## Abstract

The human glial fibrillary acidic protein (hGFAP) promoter has been used to generate numerous transgenic mouse lines, which has facilitated the analysis of astrocyte function in health and disease. Here, we evaluated the expression levels of various hGFAP transgenes at different ages in the two most commonly used inbred mouse strains, FVB/N (FVB) and C57BL/6N (B6N). In general, transgenic mice maintained on the B6N background displayed weaker transgene expression compared with transgenic FVB mice. Higher level of transgene expression in B6N mice could be regained by crossbreeding to FVB wild type mice. However, the endogenous murine GFAP expression was equivalent in both strains. In addition, we found that endogenous GFAP expression was increased in transgenic mice in comparison to wild type mice. The activities of the hGFAP transgenes were not age-dependently regulated. Our data highlight the importance of proper expression analysis when non-homologous recombination transgenesis is used.

## Introduction

Glial fibrillary acidic protein (GFAP) is the major intermediate filament protein in astrocytes, the main glia population of the brain, and has become the bona fide marker of astrocytes [1]–[3]. GFAP expression starts already during embryonic development in radial glia [3]–[5] and is highly sensitive to any kind of pathology such as acute brain injury (stroke, trauma), chronic neurodegeneration (Alzheimer’s and Parkinson’s disease) and aging [6]–[11].

In the past two decades, a 2.2 kb fragment 5′ upstream of the open reading frame of the human GFAP gene (hGFAP promoter) [12], [13] has been frequently used to drive transgenic expression of several proteins (e.g. LacZ, GFP or Cre) selectively in astrocytes [14]–[16]. To study physiological properties of astrocytes, we used this promoter for transgenic expression of fluorescent proteins (FPs) and the tamoxifen-inducible Cre DNA recombinase CreERT2 (CT2, a fusion protein of the Cre DNA recombinase and the ligand-binding domain of the estrogen receptor) [17]–[20]. Transgenic mice were generated by injection of linearized vector DNA [21] into oocytes of the most commonly used inbred mouse strains, FVB/N (FVB) and C57BL/6N (B6N). FVB mice (white fur) with large litters and high reproductive capacity are widely used for gene transfer experiments owing to their large and prominent pronuclei [22]. B6N mice (black fur) represent the preferred mouse strain for behavioral experiments despite developing spontaneous auditory degeneration in young adulthood [23]–[25].

To generate a homogenous genetic background suitable for a wide range of behavioral experiments, we crossbred transgenic FVB mice (expressing ECFP, EGFP or CT2 under the control of hGFAP promoter) to B6N mice. Unexpectedly, we found that the transgenic protein expression was strongly influenced by the genetic background of the mouse strain. Since hGFAP transgenic mice are widely used within the scientific community, we performed a quantitative comparison of transgene expression in both inbred strains.

## Materials and Methods

### Ethics Statement

This study was carried out at the University of Saarland in strict accordance with the recommendations to European and German guidelines for the welfare of experimental animals. Animal experiments were approved by the Saarland state’s “Landesamt für Gesundheit und Verbraucherschutz" in Saarbrücken/Germany (animal license number: 72/2010).

### Animals

**Figure 1:**
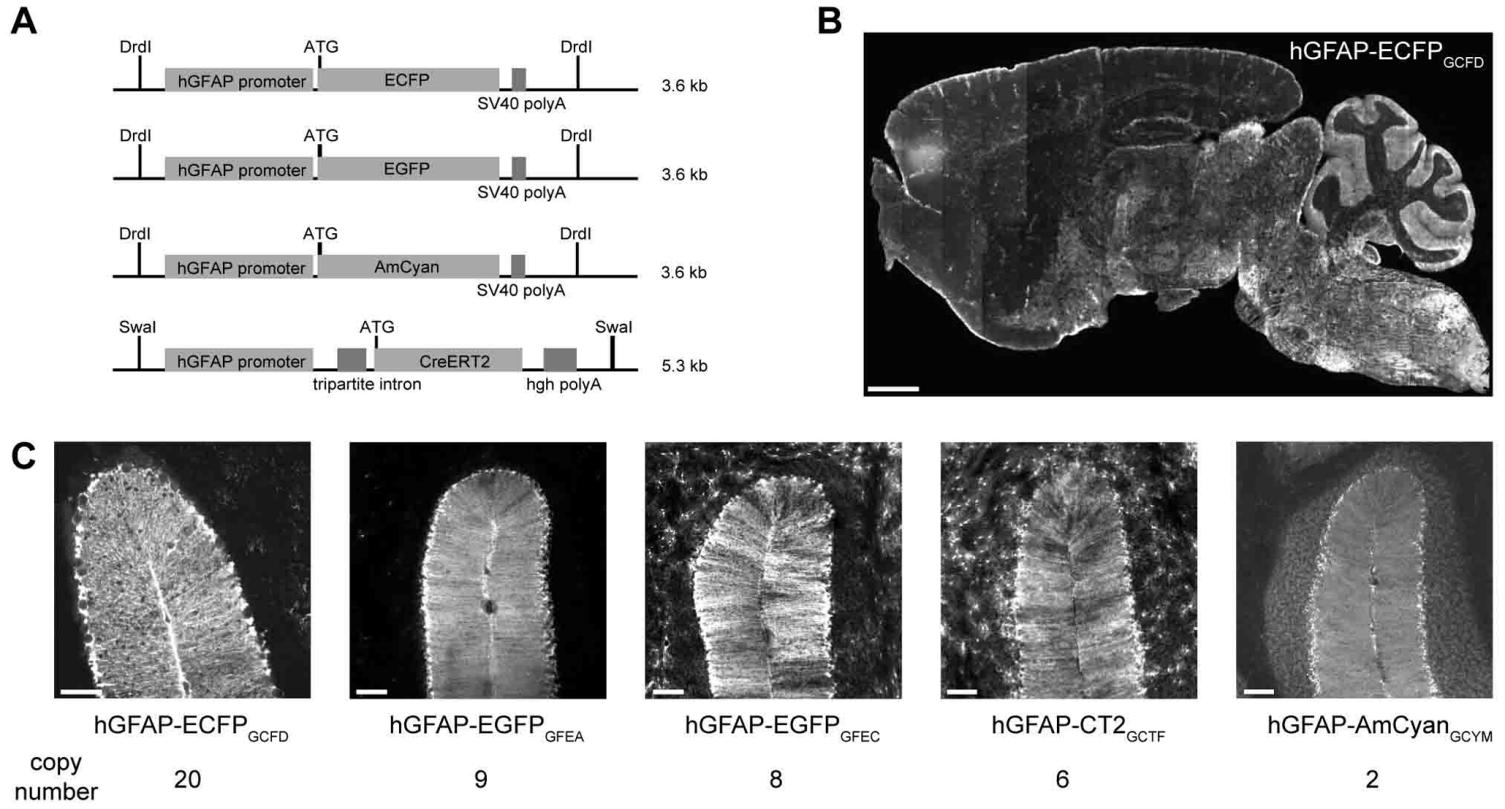
hGFAP promoter controlled transgene expression in five different mouse lines. (A) Transgenic constructs used for oocyte injection. (B) Widespread expression of ECFP in FVB(hGFAP-ECFP)_GCFD_ mice with high levels in the cerebellum. Scale bar indicates 1 mm. (C) Abundant fluorescent signals from Bergmann glia of FVB(hGFAP-ECFP)_GCFD_, FVB(hGFAP-EGFP)_GFEA/C_, B6N(hGFAP-AmCyan)_GCYM_ and FVB(hGFAP-CT2_GCFT_ × R26tdTom). Transgene copy numbers are indicated below the respective mouse lines. Scale bars indicate 100 µm.

FVB/NRj (FVB) and C57Bl/6NRj (B6N) wild type mice were used as wild type (WT) controls (purchased from Janvier, France). Transgenic mice TgN(hGFAP-ECFP)_GCFD_ = hGFAP-ECFP_GCFD_, TgN(hGFAP-EGFP)_GFEA/GFEC_ = hGFAP-EGFP_GFEA/GFEC_ and TgN(hGFAP-CreERT2)_GCTF_ = hGFAP-CT2_GCTF_ were originally generated by non-homologous recombination in the FVB background [18]–[20], expressing FPs and CT2 in astrocytes (Fig. 1A). After at least 12 generations of crossbreeding to B6N mice, they were considered as being of B6N background. TgN(hGFAP-AmCyan)_GCYM_ = hGFAP-AmCyan_GCYM_ mice were originally generated by injection of DNA in B6N oocytes [18]. We crossbred B6N(hGFAP-ECFP)_GCFD_ and B6N(hGFAP-AmCyan)_GCYM_ to FVB once to get B6NxFVB1 and twice to get B6NxFVB2 using transgenic males and wild type females (Fig. 1A). The four-letter indices represent distinct founder lines. For visualization of recombined cells in hGFAP-CT2_GCTF_, mice were bred to TgH(Rosa26-CAG-loxP-stop-loxP-tdTomato) (R26tdTom) reporter mice (Jaxlab: B6; 129S6-Gt (ROSA) 26Sortm14 (CAG-tdTomato) Hze/J [26], in which CAG represents the ubiquitously active cytomegalovirus enhancer fused to the chicken beta-actin promoter. Litters of FVB (hGFAP-CT2)_GCTF_ mice are of mixed background when crossed to R26tdTom reporter mice which are in a mixed C57BL/6J C57BL/6N background.

### Real Time-PCR and Western Blot Analysis

Levels of messenger RNA (mRNA) and genomic DNA were detected by reverse transcriptase PCR (RT-PCR), levels of proteins were detected by sodium dodecyl sulfate polyacrylamide gel electrophoresis and subsequent Western blot analysis as described previously [19], [27]. The cerebellum was homogenized (Precellys homogenizer, peqlab, Erlangen, Germany) and divided for RNA extraction (1/6) with RNeasy mini kit (QIAGEN, Hilden, The Netherlands) and for protein analysis (5/6). Primer sequences for RT-PCR were as follows (in 5′ to 3′ direction): GFAP-forward, TGG AGG AGG AGA TCC AGT TC; GFAP-reverse, AGC TGC TCC CGG AGT TCT; ExFP (EGFP and ECFP)-forward, GAA GCG CGA TCA CAT GGT; ExFP-reverse, CCA TGC CGA GAG TGA TCC; AmCyan-forward, GAG AAC CTT CAC CTA CGA GGA C; AmCyan-reverse, TCG AAG CAG TTG CCC TTC; Cre-forward, CCT GGA AAA TGC TTC TGT CCG; Cre-reverse, CAG GGT GTT ATA AGC AAT CCC; β-actin-forward, GGG TCA GAA GGA CTC CTA TG; β -actin-reverse, GGT CTC AAA CAT GAT CTG GG.

After gel separation, proteins were transferred to nitrocellulose membrane and probed with polyclonal rabbit anti-GFAP (1∶1000, Dako cytomation, Glostrup, Denmark), polyclonal rabbit anti-GFP (1∶1000, abcam, Cambridge, England), polyclonal rabbit anti-human estrogen receptor α (1∶200, Santa Cruz, Santa Cruz, USA) or monoclonal mouse anti-α-Tubulin (1∶10000, Sigma, St. Louis, USA) antibodies.

### Analysis of Transgenic Copy Number

Transgene copy number was determined by quantitative RT-PCR as described previously with slight modifications [28]. Briefly, plasmids of hGFAP-ECFP, hGFAP-EGFP, hGFAP-CT2 and hGFAP-AmCyan were used to establish a copy number standard curve. Genomic DNA was extracted from respective mouse tails with the Spin Tissue Mini kit (Stratec Molecular, Berlin, Germany). We selected heterozygous and homozygous NG2-EYFP [29] and NG2-CreERT2 (provided by Wenhui Huang, unpublished) knock-in mice as copy number controls for ECFP/EGFP and CT2, respectively. For the hGFAP-AmCyan transgene, we extracted genomic DNA from primary astrocytes [30]. The primers for ExFPs, CT2 and AmCyan were the same as the one used for cDNA PCR.

### Tamoxifen Treatment

To induce DNA recombination in hGFAP-CT2_GCTF_ × R26tdTom reporter mice, tamoxifen (10 mg/ml corn oil, Sigma, St. Louis, USA) was intraperitoneally injected into seven-week-old mice for three consecutive days (100 mg/kg body weight). Ten days after the first injection, mice were perfused and analyzed.

### Immunohistochemical Analysis of Transgenic Protein Expression

After perfusion and post-fixation with 4% formaldehyde in 0.1 M phosphate buffer (pH 7.4), free-floating vibratome brain slices were generated, blocked and permeabilized as described previously [31]. Slices were incubated with polyclonal goat anti-GFP (for ECFP and EGFP, 1∶1000, Rockland, Gilbertsville, USA) and/or polyclonal rabbit anti-S100β (1∶500, abcam, Chambridge, England) followed by incubation with Alexa488-conjugated anti-goat IgG/Alexa555-conjugated anti-rabbit IgG (1∶2000, Invitrogen, Grand Island NY, USA).

### Statistical Analysis

Three animals of every experimental age group and every strain were studied in three independent experiments. In RT-PCR experiments, cerebella of pups (one week old, 1 w) and adult mice (eight weeks old, 8 w) were investigated. We compared always mice of the same gender in both backgrounds, mostly males.

Statistical differences were analyzed using the two-tailed *t*-test for two-grouped data and one-way Anova for three-grouped data. Data are shown as mean+SEM.

## Results

### Description of hGFAP Transgenic Mouse Lines

Transgenic mice used in this study were generated by non-homologous recombination with different transgene copy numbers (TCN). Their detailed expression patterns have already been described previously [17]–[20]. The transgenic mouse lines used for comparison of transgene activity are categorized in three groups (Fig. 1A): (1) hGFAP-ECFP_GCFD_ (TCN = 20) and hGFAP-EGFP_GFEA/GFEC_ (TCN = 9 and 8, respectively) are based on the same vector with a SV40 polyA site and injected into FVB oocytes. (2) hGFAP-AmCyan_GCYM_ (TCN = 2) is based on the same vector, but injected into B6N oocytes. (3) The vector to produce hGFAP-CT2_GCTF_ (TCN = 6) contained additional regulatory elements (a generic intron in front of the ATG start codon and the polyA site of the human growth hormone instead of the SV40 polyA [32]. Vector DNA was injected into FVB oocytes. In groups 1 and 2, the expression of the FPs was directly controlled by the hGFAP promoter, while the hGFAP-CT2 mouse line required crossbreeding to a Cre-reporter line. For that purpose we used the R26tdtom mouse line, in which the final expression level in astrocytes was controlled by a ubiquitously active promoter (CAG) [26].

All transgenic mice were fertile and could be crossed to homozygosity without overt pathological phenotype. Genetically modified mice, generated by non-homologous recombination, are known for line-dependent transgene expression patterns [33], [34]. In the CNS of our mouse lines we also observed a region dependent pattern of transgene expression. Only 10 to 30% of cortical astrocytes expressed ECFP, while 60 to 90% of all Bergmann glia in the cerebellum and astrocytes in the brainstem expressed ECFP in the hGFAP-ECFP_GFCD_ mouse line (Fig. 1B). However, within the progeny of a given line, in the same inbred strain, the expression pattern did not change.

To evaluate the impact of genetic background on transgene expression, we focused on Bergmann glia (Fig. 1C) in the following lines: hGFAP-ECFP_GCFD_, hGFAP-EGFP_GFEC_, hGFAP-CT2_GCTF_ and hGFAP-AmCyan_GCYM_. The highly organized distribution of Bergmann glia facilitated the quantitative analysis by cell-counting.

We analyzed the sensitivity of the hGFAP promoter (gfa2) [12], [13] in FVB and B6N strains by comparing protein and mRNA levels.

### Transgenic Mice in B6N Backgrounds Displayed Diminished Transgene Expression

**Figure 2:**
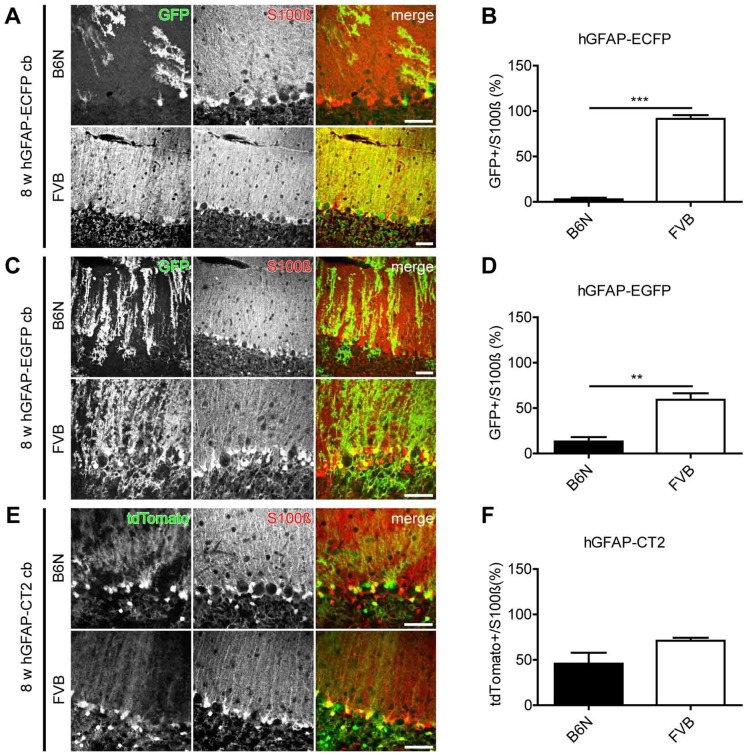
Immunohistochemical analysis of reporter protein expression in different transgenic mouse lines showed lower expression in the B6N background when compared to FVB. Cerebellar vibratome slices (cb) of 8-week-old mice were immunolabeled with anti-GFP (A and C) and anti-S100β antibodies (A, C and E), endogenous fluorescence of tdTomato in E. Upper panels depict transgene expression in B6N, lower panels in FVB. The S100β staining indicates all Bergmann glia. Results of comparative analysis in B6N and FVB mice are presented as percentage of transgene expressing Bergmann glia (S100β positive cells) (B, D and F). ***: p<0.001, **: p<0.01. Scale bars indicate 50 µm.

**Figure 3:**
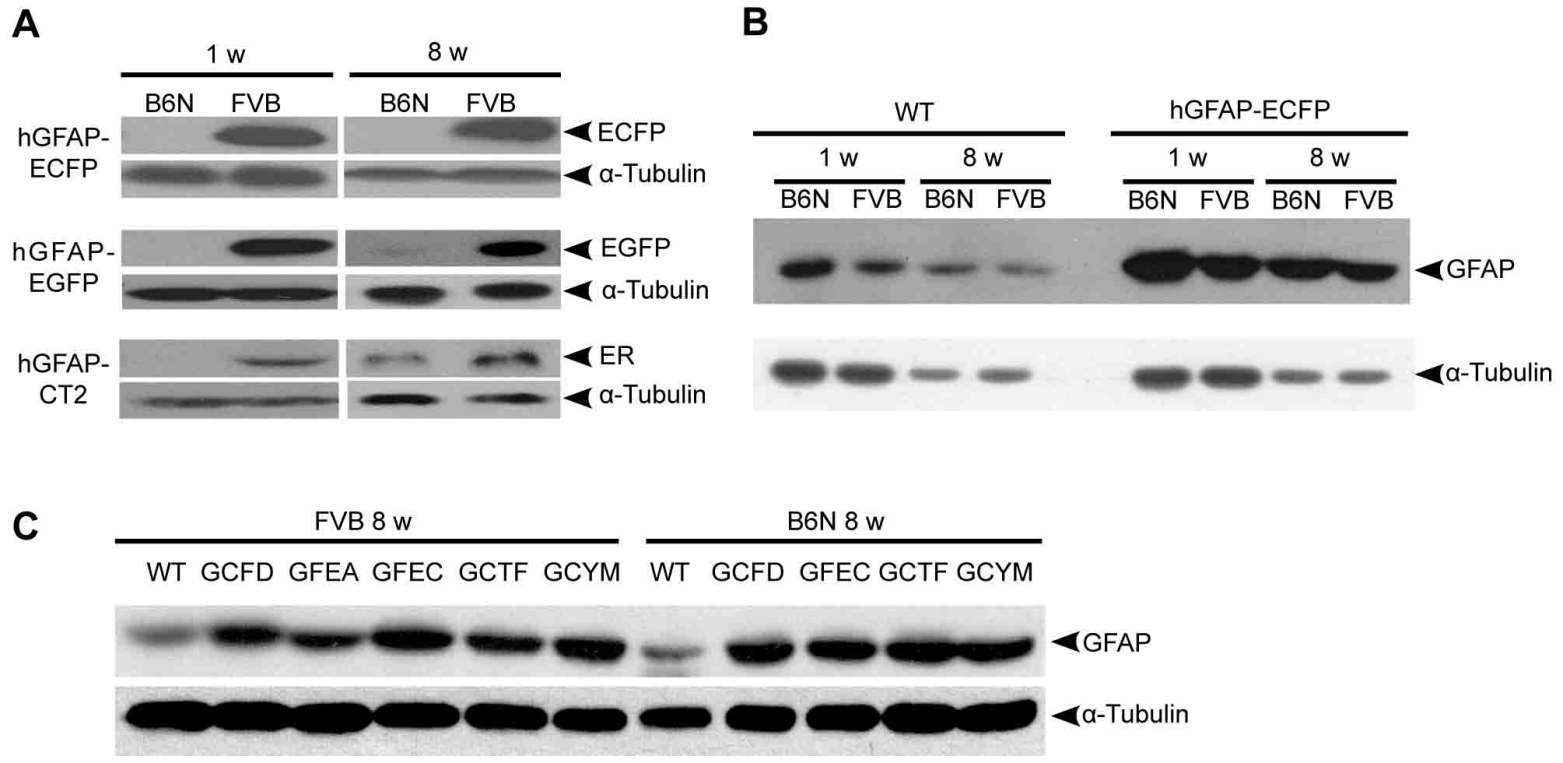
Comparative Western blot analysis of endogenous GFAP and transgenic proteins in B6N and FVB mice. Cerebellar homogenates of transgenic and wild type mice (1 w and 8 w) were probed with anti-GFP (to detect ECFP or EGFP), anti-human estrogen receptor α (ER α, recognizing CT2), and anti-GFAP and anti-α-tubulin antibodies. (A) Western blot analysis of transgene expression. (B) Western blot analysis of endogenous GFAP expression in WT and transgenic mice (hGFAP-ECFP)_GCFD_. (C) Western blot analysis of endogenous GFAP expression in WT and five transgenic mouse lines (hGFAP-ECFP_GCFD_; hGFAP-EGFP_GFEA_; hGFAP-EGFP_GFEC_; hGFAP-CT2_GCTF_; hGFAP-AmCyan_GCYM_) in both FVB and B6N background.

The extent of FP expression (ECFP and EGFP) in FVB and B6N mice was evaluated by cell counting after immunohistochemistry (Fig. 2) and Western blot analysis (Fig. 3).

To compensate for differences in physical fluorescence properties we enhanced the signal by using anti-GFP antibodies for both hGFAP-ECFP_GCFD_ and hGFAP-EGFP_GFEC_ mouse lines in FVB and B6N backgrounds. Immunohistochemistry data revealed that nearly all Bergmann glial cells expressed ECFP (91.5±4.0%) (Fig. 2A and B) in FVB(hGFAP-ECFP)_GCFD_ mice, however, ECFP was hardly detectable in B6N(hGFAP-ECFP)_GCFD_ mice (2.8±1.8%, Fig. 2A and B). A reduction in transgene expression was also observed in another transgenic mouse line: in FVB(hGFAP-EGFP)_GFEC_, in which 59.2±7.1% of Bergmann glia were EGFP-positive, while markedly less EGFP expressing Bergmann glia (13.2±4.9%) were observed in B6N(hGFAP-EGFP)_GFEC_ mice (Fig. 2C and D). We further analyzed a third transgenic mouse line, hGFAP-CT2_GCTF_, in which the inducible Cre DNA recombinase CT2 was expressed under the control of the same hGFAP promoter. To activate CT2, we injected tamoxifen to hGFAP-CT2 × R26tdTom female mice for three consecutive days to induce recombination and subsequent expression of the red fluorescent reporter protein tdTomato in astrocytes. Ten days after the first tamoxifen injection, reporter expression was not significantly different in FVB mice (71.1±3.2%) compared to B6N mice (45.8±12.0%) (Fig. 2E and F).

We confirmed the higher expression of FPs and CT2 in cerebella of FVB mice by Western blot analysis. Young FVB mice showed significantly higher expression of transgenes than B6N mice in all three examined lines (left panel in Fig. 3A) (ratios: FVB_ECFP_/B6N_ECFP_ = 37.3; FVB_EGFP_/B6N_EGFP_ = 8; FVB_CT2_/B6N_CT2_ = 2.81). No or only a weak protein signal could be detected in B6N(hGFAP-ECFP)_GCFD_ and B6N(hGFAP-EGFP)_GFEC_ adult mice. In contrast, in B6N(hGFAP-CT2)_GCTF_ adult mice CT2 expression was clearly detectable (Fig. 3A) but still significantly lower compared to FVB mice.

We then investigated whether the endogenous GFAP expression varies in WT and transgenic mouse lines. The expression of the hGFAP transgenes appeared to be independently regulated from the endogenous mouse GFAP gene (Fig. 3B). In WT as well as in hGFAP-ECFP_GCFD_ mice the level of endogenous GFAP was not different between B6N and FVB mice of the same age. However, we found that WT mice expressed less GFAP than transgenic mice in all the transgenic mouse lines that we studied in this work (Fig. 3C).

Cell counting after immunohistochemical labeling as well as Western blot analysis revealed higher levels of FP expression in FVB compared to B6N mice. CT2 protein expression was significantly higher in FVB cerebellar homogenates; however, tdTomato reporter expression analysis was similar in transgenic FVB and B6N mice.

### Lower Transgenic FP and CT2 mRNA Levels in B6N than in FVB Mice

**Figure 4:**
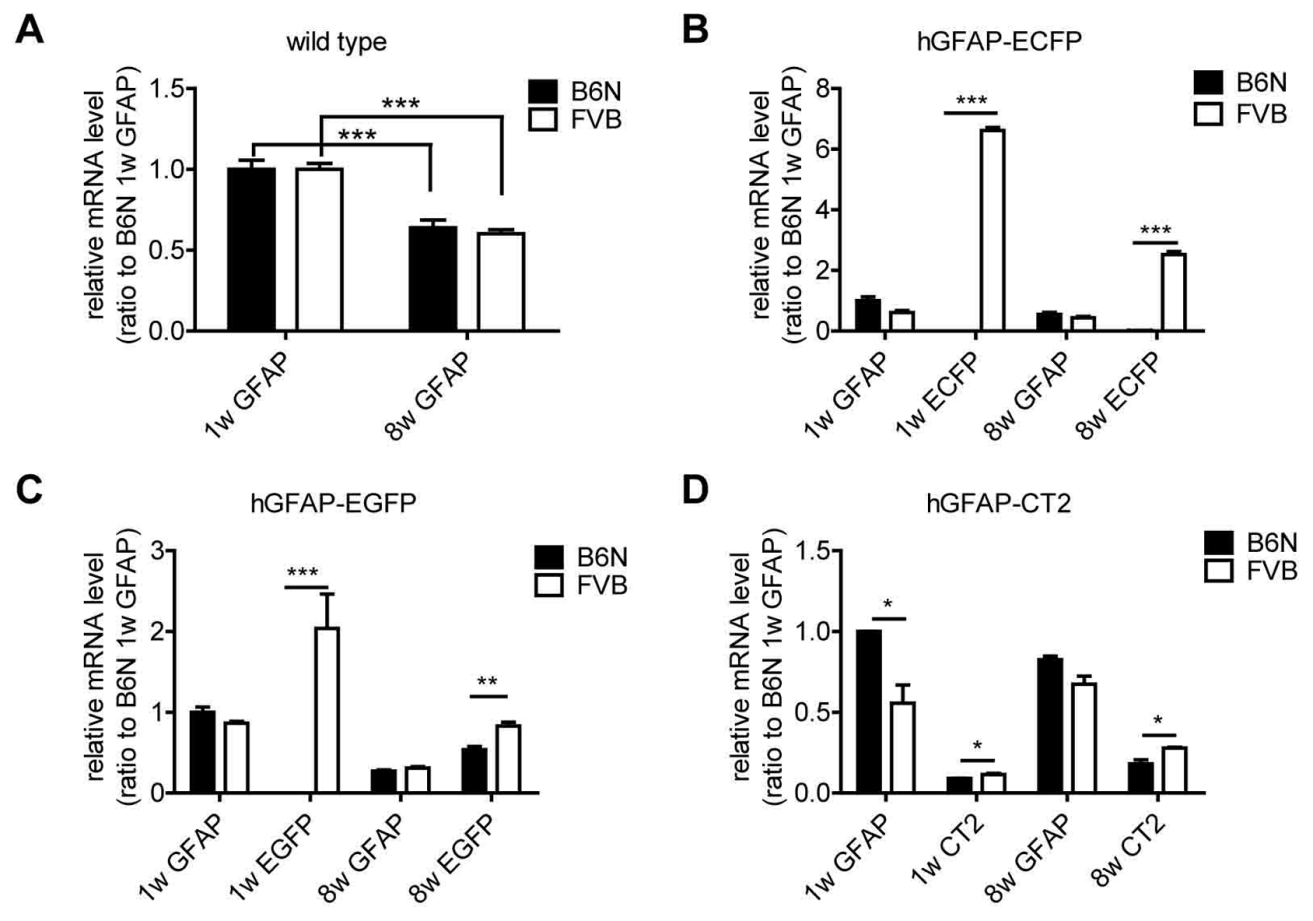
Quantitative RT-PCR analysis of transgene and endogenous GFAP mRNA levels in FVB and B6N mice. (A) Cerebellar GFAP mRNA levels in wild type B6N and FVB mice (1 w and 8 w). (B-D) Transgenic mRNA levels compared to endogenous GFAP mRNA levels in the cerebellum of B6N and FVB mice (1 w and 8 w). (B) hGFAP-ECFP_GCFD_. (C) hGFAP-EGFP_GFEC_. (D) hGFAP-CT2_GCTF_. Relative expression is normalized to GFAP mRNA level in 1 w B6N mice. *: p<0.05, **: p<0.01, ***: p<0.001. Data are obtained from three independent experiments with samples from three mice (n = 3) in every experiment.

To investigate whether the different expression levels of transgenic proteins were caused by transcriptional/posttranslational regulation or protein degradation, we studied mRNA levels in WT and transgenic FVB and B6N mice. Both B6N and FVB WT mice showed equal levels of endogenous GFAP mRNA at the same age, consistent with the protein data (Fig. 3B). However, endogenous GFAP mRNA levels dropped significantly from young (1 w) to adult (8 w) WT mice (B6N vs. FVB: 1.0±0.06 vs. 1.0±0.04 at 1 w; 0.64±0.05 vs. 0.60±0.03 at 8 w) (Fig. 4A). After comparing the endogenous mouse GFAP mRNA levels in WT mice, we quantified mRNA levels of FPs and CT2 controlled by transgenic hGFAP promoters in both inbred strains. In line with the protein data, almost no ECFP mRNA was detectable in B6N(hGFAP-ECFP)_GCFD_ mice at any age (Fig. 4B). Also only low levels of EGFP mRNAs were detected in B6N(hGFAP-EGFP)_GFEC_ young mice, however, those increased in the adult (Fig. 4C), again consistent with our Western blot data (Fig. 3A). CT2 mRNA levels were significantly lower in B6N compared to FVB at both ages (Fig. 4D), also consistent with the protein data from the Western blots (Fig. 3A).

In young hGFAP-ECFP_GCFD_ mice (with the highest difference in protein levels), ECFP mRNA levels were about 3500 times higher in FVB than in B6N (Fig. 4B), however, in hGFAP-CT2_GCTF_ mice of the same age, the CT2 mRNA levels were almost comparable (FVB/B6N = 1.25, Fig. 4D). Furthermore, ECFP was down-regulated with age in FVB(hGFAP-ECFP) (37% remaining, Fig. 4B), while the other two lines displayed either similar (FVB(hGFAP-EGFP_GFEC_), Fig. 4C) or increased (B6N(hGFAP-EGFP_GFEC_ and hGFAP-CT2) and FVB(hGFAP-CT2), Fig. 4C and D) levels of mRNA in adult mice.

Additionally, one crossbreeding of the second EGFP-expressing transgenic line, FVB(hGFAP-EGFP)_GFEA_ (Fig. 1C), to B6N WT mice significantly reduced EGFP mRNA level in FVBxB6N1 littermates compared with FVB mice (FVB:FVBxB6N1 ratio = 6.75), while endogenous GFAP mRNA levels were equal as shown for the other transgenic lines before (data not shown).

Taken together, quantitative RT-PCR results confirmed that FVB mice showed higher hGFAP promoter activity as confirmed by higher transgene mRNA level. However, the endogenous GFAP mRNA levels were equal among all the WT and transgenic mouse lines except in hGFAP-CT2 line.

We also noted that the activity of the hGFAP promoter is highly variable. For instance, in young FVB(hGFAP-ECFP)_GCFD_ mice the ECFP mRNA levels were about 11 times higher than the endogenous GFAP mRNA levels, while in young FVB(hGFAP-CT2)_GCTF_ transgenic mice CT2 mRNA levels were decreased to 18.5% (Fig. 4B–D).

### Crossbreeding of B6N Mice to FVB Increases Transgene Expression

**Figure 5:**
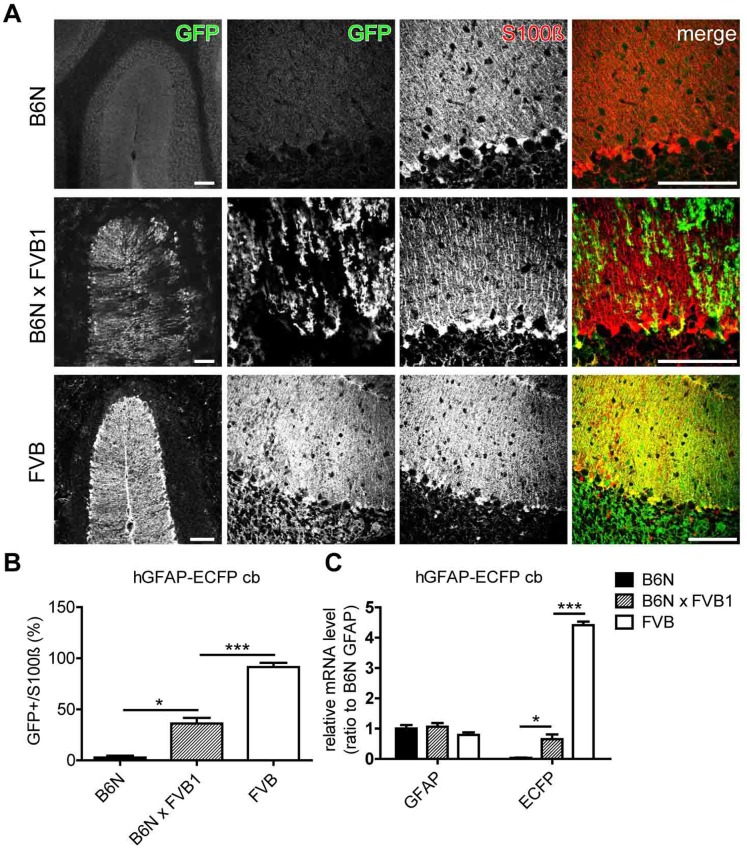
Backcrossing of B6N (hGFAP-ECFP)GCFD mice to FVB for a single generation re-activated transgenic ECFP expression. (A and B) Cerebellar slices of 8-week-old mice were immunostained with anti-GFP and anti-S100β antibodies and analyzed. Only single ECFP expressing Bergmann glia (S100β positive cells) were detected in B6N(hGFAP-ECFP)_GCFD_ mice (A, upper panel), while ∼91.5% of Bergmann glia were ECFP positive in FVB(hGFAP-ECFP)_GCFD_ mice (A, lower panel). Backcrossing of B6N(hGFAP-ECFP)_GCFD_ for one generation with FVB WT mouse led to increased ECFP expression in B6NxFVB1 littermates (A, middle panel). (C) GFAP and ECFP mRNA levels in B6N, FVB and B6NxFVB1 mice (8 w). Relative expression is normalized to GFAP mRNA level in B6N mice. *: p<0.05 and ***: p<0.001. Data are obtained from three independent experiments with samples from three mice (n = 3) in every experiment. Scale bars indicate 100 µm.

So far we could demonstrate that crossbreeding FVB transgenic mice to B6N results in a severe down-regulation of transgene expression. Therefore, we wanted to know whether the reverse experiment, backcrossing of transgenic B6N mice to FVB, could enhance low transgene expression (Fig. 5). For this purpose, we selected the hGFAP-ECFP_GCFD_ mouse line, because it exhibited the strongest difference in transgene expression between B6N and FVB (Fig. 2A, B and 5A). Strikingly, one single backcrossing (B6NxFVB1) already significantly reactivated ECFP mRNA 22-fold (B6N vs. B6NxFVB1: 0.03±0.01 vs. 0.65±0.16) and protein expression 13-fold (B6N vs. B6NxFVB1: 2.8±1.8% vs. 36.2±5.5%) in B6NxFVB1 mice compared to B6N (Fig. 5). These results again indicate that FVB mice have higher hGFAP promoter activity than B6N. This observation provides additional proof that the genetic background has a clear impact on hGFAP promoter activity.

**Figure 6:**
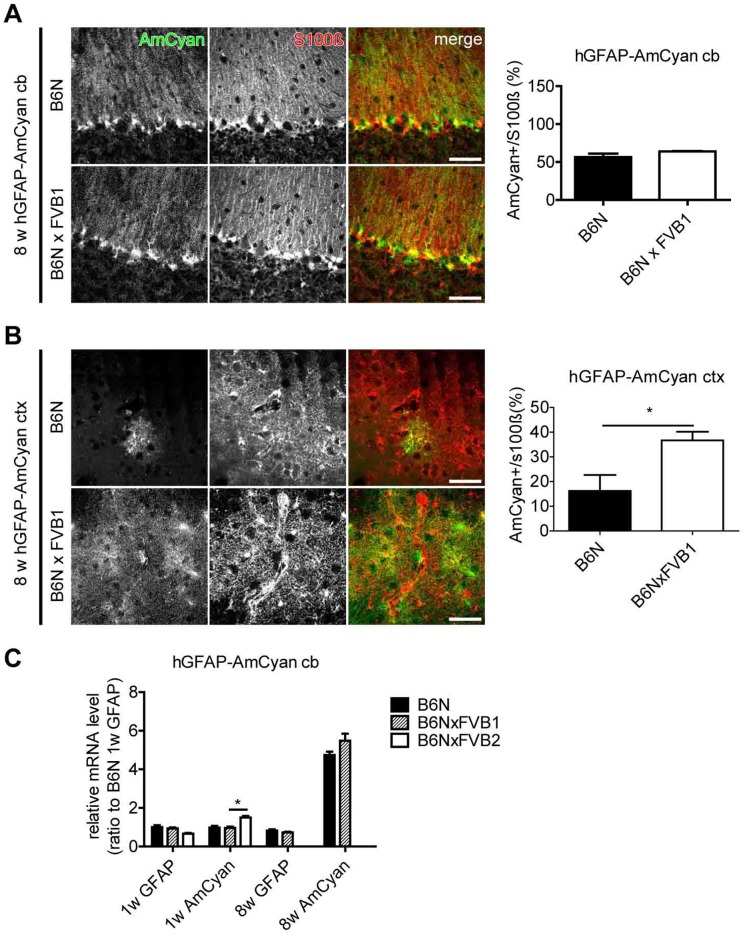
Backcrossing of B6N(hGFAP-AmCyan)GCYM mice to FVB increased transgenic AmCyan expression. Cerebellar (A) and cortical (B) brain slices of 8-week-old mice were immunostained with anti-S100β antibodies. Backcrossing of B6N(hGFAP-AmCyan)_GCYM_ (upper panels in A and B) for one generation to FVB (lower panels in A and B) did not significantly enhance AmCyan expression in cerebellum, but caused higher levels in the cortex of B6NxFVB1 littermates when compared to B6N. Results of comparative analysis (right panels in A and B) in B6N and B6NxFVB1 mice are provided as percentage of transgene expressing Bergmann glia (A) and cortical astrocytes (B) (S100β positive cells). (C) GFAP and AmCyan mRNA levels in 1-week-old (B6N, B6NxFVB1 and B6NxFVB2) and 8-week-old (B6N and B6NxFVB1) mouse cerebellum. A second backcrossing resulted in enhanced transgene levels. Relative expression is normalized to GFAP mRNA level in 1-week-old B6N mice. *: p<0.05. Data are obtained from three independent experiments with samples from three mice (n = 3) in every experiment. Scale bars indicate 50 µm.

We further tested the effect of backcrossing to FVB in an additional mouse line. But this time we chose a mouse line, i.e. hGFAP-AmCyan_GCYM_, which was originally generated by injection of the transgenic vector into B6N oocytes and maintained in a B6N background. After crossing for only one generation to FVB (Fig. 6A) we realized that AmCyan expression in the cerebellum, e.g. the number of reporter-positive Bergmann glia, was not significantly enhanced (B6N vs. B6NxFVB1: 56.6±4.5 vs. 64.0±0.5%). The mRNA levels of AmCyan were comparable as well (B6N vs. B6NxFVB1: 0.98±0.09 vs.0.96±0.07). An additional backcrossing (B6NxFVB2) showed a significantly higher level of AmCyan mRNA when compared to B6N and B6NxFVB1 (B6NxFVB2: 1.5±0.08) (Fig. 6C).

However, when analyzing the cortex, a brain region where AmCyan expression levels were initially very low (16.2±6.4%), we could detect a more than two-fold increase of AmCyan-expressing astrocytes already in B6NxFVB1 (36.7±3.4%) when compared to B6N mice (Fig. 6B).

These results suggest that a low hGFAP promoter activity in B6N mice can be increased by crossbreeding to the FVB background.

## Discussion

In the current study, we investigated in different transgenic mouse lines the hGFAP promoter-controlled expression of FPs or CT2. We found that the activity of this promoter was strongly dependent on the chosen inbred strain, FVB or B6N.

### (1) Transgene Activity in Inbred Strains

FVB mice expressed higher FP and CT2 levels than B6N, especially the hGFAP-ECFP_GCFD_ and hGFAP-EGFP_GFEC_ lines (Fig. 2, 3 and 4). In addition, a single backcrossing of B6N to FVB rescued silenced FP expression (Fig. 5) or increased the existing expression (Fig. 6). All our data demonstrate a stronger activity of the hGFAP promoter in FVB than in B6N mice.

Three different mechanisms have been reported to regulate the transcription of the *Gfap* gene: DNA methylation; histone methylation and acetylation; as well as spatial positioning.

### DNA Methylation Influences Transcription Factor Binding

Epigenetic studies showed that in early stages of embryonic development, the methylation of the GFAP promoter at CpG islands represses transcription by preventing the binding of STAT3 (signal transducer and activator of transcription 3) in a complex with Smad1/4 (signal transducer and transcriptional modulator) and p300 at the corresponding promoter element [35]–[37]. During late embryogenesis, enhanced demethylation of the GFAP promoter and subsequently increased GFAP expression are characteristic properties of astroglial differentiation [36]. Similarly, in human malignant gliomas, the GFAP expression is controlled by methylation. Here, however, an enhanced methylation of the promoter causes a silencing of the *Gfap* gene [38]. Similarly, for the imprinted transgene RSVIgmyc higher levels of methylation were found in C57BL/6J than in FVB [39], indicating higher methylation activities in B6N. Since the *Gfap* gene transcription occurs monoallelically in the cerebral cortex [40], different methylation conditions could be a very potent mechanism to cause the observed different transgene expression levels in B6N and FVB.

### Histone Methylation and Acetylation Affect Chromatin Structure

Similar to DNA methylation, histone methylation represents another mechanism of transcriptional silencing or activation. Growth factors (basic fibroblast growth factor 2) positively affect the binding of the STAT/CBP complex with the GFAP promoter by inducing H3K4 (lysine 4 at histone 3) methylation and suppression of H3K9 (lysine 9 at histone 3) methylation around the STAT3-binding site, leading to an increased GFAP expression in developing astrocytes [37]. Histone acetylation can be positively related to transcriptional activity as well [41]. At the GFAP promoter, binding of STAT3 to the CBP/p300 complex activates the intrinsic histone acetyltransferase of the coactivators CBP and p300 and subsequent relaxing of the chromatin structure, resulting in enhanced transcription [42]. Unfortunately, the GFAP promoter difference in histone acetylation/methylation between inbred strains has not yet been investigated.

### Spatial Positioning as a Mean to Regulate Transcription

The spatial positioning of gene loci within the nucleus has been discussed as a mechanism of transcriptional regulation. In cultured astrocytes the active *Gfap* alleles appear preferentially positioned towards the center of the nucleus while inactive alleles are more frequently found at the periphery as it could be shown by fluorescence *in situ* hybridization [40], [41]. Transcription-preferring localization within the nuclear architecture appears as an effective mean to regulate gene expression, and it is tempting to speculate that such chromatin remodeling mechanisms are subject to the genetic background of inbred strains.

### (2) Differences in Transgenic Constructs

The variable composition of the transgenic plasmids used to generate the analyzed mouse lines, seems to affect the transgenic expression pattern. For hGFAP-ECFP_GCFD_, hGFAP-EGFP_GFEA/GFEC_ and hGFAP-AmCyan_GCYM_ lines, the simplest cloning strategy has been used: A fragment of the hGFAP promoter (gfa2) [12], a Kozak sequence (TCG CCA CCA TG, [43]) followed by the open reading frame (ORF) of the transgenic protein and termination by the SV40 polyadenylation (polyA) sequence [44], [45] (Fig. 1C). For the generation of hGFAP-CT2 transgenic mice, the construct was modified by insertion of a generic intron to stabilize primary transcripts [46]. In addition, the viral polyA sequence was exchanged with an eukaryotic polyA sequence (hgh polyA, human growth hormone, [32]). For transgenesis several hundred linearized DNA molecules were injected into a single fertilized oocyte, which usually integrate as concatemers into the genome [47].

The protein and mRNA data suggest that CT2 mice are less affected by the change of inbred strains compared with FP-transgenic mice (hGFAP-ECFP_GCFD_ and hGFAP-EGFP_GFEC_). The levels of expressed CT2 protein and mRNA were still lower in B6N than FVB, but the overall difference was strikingly lower than in the lines with FP expression (Fig. 2, 3 and 4). Since splicing is known as an mRNA stabilizing mechanism [47]–[49], we assume that the additional splicing induced by the generic intron in the CT2 construct reduces the transcriptional variability between the inbred strains as prominently observed with the FP constructs. In addition, the recombination frequency (the functional readout of the CT2 enzyme activity) was not affected by the genetic background. Besides the improved stability of the mRNA this might also be due to the low number of enzyme molecules that are required for recombination of loxP sites and resulting in reporter protein expression (tdTomato) in FVB and B6N after CT2 induction (Fig. 2E and F).

### (3) Transgene Copy Number

Previous reports have shown that transgene copy number (TCN) affects the level of transgene expression in the mammalian system due to the concatemeric integration [50]. While lower copy numbers lead to higher transgene expression, high copy numbers have the opposite effect. Here, we observed that mouse lines with higher TCN (hGFAP-ECFP_GCFD_ = 20 copies) showed an overall high transgene expression (FVB: more than 90% of Bergmann glia) compared to mouse lines with smaller TCN (hGFAP-EGFP_GFEC_ = 8 copies) (FVB: about 60% of Bergmann glia). Compared to background changes the mouse lines with higher TCN have shown higher sensitivities to inbred strain changes while mouse lines with smaller TCN were less sensitive (Fig. 3A). For hGFAP-AmCyan_GCYM_ (2 copies) this could additionally explain why we could not detect a significant difference in Bergmann glial AmCyan expression after a single backcross to FVB (B6N vs. B6NxFVB1: both around 60%, Fig. 6). In contrast, crossing of B6N(hGFAP-ECFP)_GCFD_ mice to FVB led to a remarkable 13-fold increase.

However, also the design of the construct can reduce the impact of TCN on transgenic protein expression or transgenic mRNA levels. This could be shown by the CT2 construct, where the differences between B6N and FVB were remarkably smaller than in the FP lines (Fig. 3 and 4) while the copy numbers were comparable (hGFAP-EGFP_GFEC_ = 8 and hGFAP-CT2_GCTF_ = 6).

### (4) Endogenous GFAP

In all analyzed transgenic mouse lines the Western Blot analysis of cerebellar homogenates indicated an upregulation of endogenous GFAP protein (Fig. 3C) compared with both WT strains. However, further analysis by qPCR revealed no difference in endogenous GFAP promoter activity: the mRNA levels did not change between the background strains. Also the number of GFAP positive cells was comparable in B6N and FVB (data not shown). Previous studies using hGFAP transgenes did not report an upregulation of the endogenous GFAP level [4], [5], [17]–[19], [51]–[57]. We assume that the increase in GFAP protein might be harder to detect when using immunofluorescence detection techniques that are most frequently exerted. Although the increase in GFAP could be an early indicator of a slight pathology, we did not observe behavioral abnormalities in our mouse lines [17]–[19].

### (5) Developmental Regulation of GFAP

GFAP mRNA expression is developmentally regulated. Endogenous mRNA levels peak at the first and second postnatal week and decrease into adulthood [58], [59], an observation we could confirm in WT mice (Fig. 4A). However, transgenic mRNAs were differently regulated, with decreases of FP mRNAs in FVB(hGFAP-ECFP)_GCFD_ and FVB(hGFAP-EGFP)_GFEC_ in line with the endogenous GFAP mRNA, while mRNA levels of the FP in B6N(hGFAP-EGFP)_GFEC_ and of CT2 in both backgrounds of hGFAP-CT2_GCTF_ increased with age (Fig. 4D and 6C), thereby indicating the presence of different regulatory mechanisms.

### Conclusion

The random transgene insertion site underlies local influences of cis-acting regulatory elements that thereby affect the strength of transgenic expression and the high variability of expression patterns among individual founders [33], [34], [47], [60], [61]. Additionally, the copy number of transgene insertion could influence the stability of transgenic expression [50]. Here, we show that also changing the inbred strain strongly modulates the activity of the human GFAP promoter. FVB mice showed always higher transgenic activity than B6N mice at the same age. By extended crossing into the FVB or B6N background and vice versa the level of transgene expression could be reversibly (in the time span of generations) modulated.

Although all our mouse lines showed weaker expression in B6N, it is hard to extrapolate whether this occurs in all hGFAP mouse lines. Since this promoter is frequently used to study astrocyte function, we recommend a careful control of the genetic background.
